# L-Arginine and Asymmetric Dimethylarginine Are Early Predictors for Survival in Septic Patients with Acute Liver Failure

**DOI:** 10.1155/2012/210454

**Published:** 2012-05-06

**Authors:** Thorsten Brenner, Thomas H. Fleming, Claudia Rosenhagen, Ute Krauser, Markus Mieth, Thomas Bruckner, Eike Martin, Peter P. Nawroth, Markus A. Weigand, Angelika Bierhaus, Stefan Hofer

**Affiliations:** ^1^Department of Anesthesiology, University of Heidelberg, Im Neuenheimer Feld 110, 69120 Heidelberg, Germany; ^2^Department of Medicine I and Clinical Chemistry, University of Heidelberg, Im Neuenheimer Feld 410, 69120 Heidelberg, Germany; ^3^Department of General and Transplant Surgery, University of Heidelberg, Im Neuenheimer Feld 110, 69120 Heidelberg, Germany; ^4^Institute of Medical Biometry and Informatics, University of Heidelberg, Im Neuenheimer Feld 305, 69120 Heidelberg, Germany; ^5^Department of Anesthesiology and Intensive Care Medicine, University of Gießen, Rudolf-Buchheim-Strasse 7, 35392 Gießen, Germany

## Abstract

Dysfunctions of the L-arginine (L-arg)/nitric-oxide (NO) pathway are suspected to be important for the pathogenesis of multiple organ dysfunction syndrome (MODS) in septic shock. Therefore plasma concentrations of L-arg and asymmetric dimethylarginine (ADMA) were measured in 60 patients with septic shock, 30 surgical patients and 30 healthy volunteers using enzyme linked immunosorbent assay (ELISA) kits. Plasma samples from patients with septic shock were collected at sepsis onset, and 24 h, 4 d, 7 d, 14 d and 28 d later. Samples from surgical patients were collected prior to surgery, immediately after the end of the surgical procedure as well as 24 h later and from healthy volunteers once. In comparison to healthy volunteers and surgical patients, individuals with septic shock showed significantly increased levels of ADMA, as well as a decrease in the ratio of L-arg and ADMA at all timepoints. In septic patients with an acute liver failure (ALF), plasma levels of ADMA and L-arg were significantly increased in comparison to septic patients with an intact hepatic function. In summary it can be stated, that bioavailability of NO is reduced in septic shock. Moreover, measurements of ADMA and L-arg appear to be early predictors for survival in patients with sepsis-associated ALF.

## 1. Introduction

Septic shock as well as the resulting MODS represent an ongoing challenge in intensive care units [1]. The pathogenesis of MODS in patients with septic shock is a multifactorial process. Recent studies have provided evidence that an impaired NO homeostasis might play an important role [2–4]. Dysfunctions of the L-arg/NO pathway have been reported to be a reason for the deleterious vascular effects of diabetes mellitus, hypercholesterolemia, hypertension, smoking, and others [5]. Moreover, reduced endothelial NO bioavailability has suspected to be crucial in patients with sepsis, since microcirculatory blood flow is disrupted and key bacterial functions in the host might be compromised [2, 4].

NO is synthesized from the conditionally essential amino acid L-arg by the action of NO-synthases (NOS) [6]. However, earlier published articles dealing with plasma levels of L-arg in septic patients revealed conflicting results [7–18]. ADMA represents one further main component of the NO homeostasis, due to its ability to serve as an endogenous NOS-inhibitor. The accumulation of ADMA was suspected to play an important role in the development of MODS and was independently associated with mortality in unselected critically ill patients [19, 20]. Competitive leveling of L-arg and ADMA with regard to NOS activity was entitled L-arginine-paradox, describing an ADMA-induced right-shift of the NOS concentration-response curve for L-arg. Only small changes in L-arg plasma concentrations may, therefore, cause marked changes in NOS activity in order to avoid a relevant lack of NO [21].

The aims of this study were, therefore, threefold: (1) to determine plasma concentrations of L-arg and ADMA as well as the resulting ratio of both in patients with septic shock, surgical patients undergoing major abdominal surgery, as well as healthy volunteers, (2) to investigate the prognostic role of each parameter in patients with septic shock, and (3) to assess the applicability of newly developed ELISA-based measurements of L-arg and ADMA.

## 2. Materials and Methods

This observational clinical study was approved by the local ethics committee (Trial-Code-Nr.: S123-2009) and was conducted in the surgical intensive care unit of the University Hospital of Heidelberg, Germany. Study and control patients or their legal designees signed written informed consent. In total, 120 patients in 3 groups were enrolled in the study. The 3 groups included 60 patients with septic shock (referred to as the septic group), 30 patients after major abdominal surgery due to a tumorous disease (the surgical group), and 30 healthy volunteers (the healthy group, Table 1). The 60 patients were classified as having septic shock based on the criteria of the International Sepsis Definitions Conference [22]. Since patients with septic shock were recruited on a surgical intensive care unit, all of them underwent a surgical procedure in varying degrees of time prior to sepsis onset. Patients were eligible for enrollment with an onset of sepsis syndrome ≤24 hours. The initial blood draw was also performed within this period. In contrast, patients with an onset of sepsis syndrome >24 hours were excluded from the study. The management of patients with septic shock in the intensive care unit included early goal-directed therapy (according to Rivers and colleagues [23]), elimination of the septic focus, and broad-spectrum antibiotics [24, 25]. Patients with renal disorders (as indicated by a serum-creatinine ≥1.2 mg/dL or 20.5 *μ*mol/L, according to sequential organ failure assessment (SOFA)-score) as well as liver diseases (as indicated by a serum-bilirubin ≥1.2 mg/dL or 20.5 *μ*mol/L, according to SOFA-score) prior to the onset of sepsis were excluded from the study. Blood samples from patients with septic shock were collected at sepsis onset (Onset), and 24 hours (24 h), 4 days (4 d), 7 days (7 d), 14 days (14 d), and 28 days (28 d) later. Relevant baseline data (demographic data, primary site of infection, outcome) and clinical data (disease severity scoring, hemodynamic data, need for catecholaminergic support, ventilator settings, etc.) were collected. Patients with septic shock were reevaluated for survival 28 days after enrollment in the study. These evaluations were performed using available hospital records. In case of the patient's discharge from the hospital, the family doctor was contacted. If necessary, direct contact was also made with the patient. Blood samples from the surgical group were collected prior to surgery (Pre), immediately after the end of the surgical procedure (Onset), and 24 hours afterwards (24 h). No later blood draws were performed in the surgical group, since peak plasma levels of proinflammatory and anti-inflammatory cytokines are reported to appear on the 1st postoperative day in surgery-induced inflammation and the investigation associated burden of the individual study patient was demanded to be as minimal as possible [26]. Blood samples from the healthy group were collected once. In order to avoid diet-related affections of L-arg or ADMA plasma levels, neither patients in the septic group, nor surgical patients received immune-enhancing nutrition.

After blood collection, plasma of all study participants was immediately obtained by centrifugation, transferred into cryotubes, and stored at −80°C until further processing. Serum tests for routine laboratory parameters (creatinine, urea, bilirubin, leukocytes, C-reactive protein, etc.) were performed at the same time.

Measurements of ADMA as well as L-arg were performed using ELISA-kits (Immundiagnostik, Bensheim, Germany) according to the manufacturer's instructions.

The resulting study data was evaluated using SPSS (statistical product and services solutions) software (Version 19.0, SPSS Inc, Chicago, USA). Categorical data were summarized by means of absolute and relative frequencies (counts and percentages). Quantitative data were summarized using the number of observations, mean, and standard deviation, as well as median with quartiles. Wherever appropriate, data were visualized using line charts or Box-and-Whisker plots. The Kolmogorov-Smirnov test was applied to check for normal distribution. Due to nonnormally distributed data, nonparametric methods for evaluation were used (chi-square test for categorical data, Mann-Whitney test for continuous data). A receiver operating characteristic (ROC) curve was established with suitable parameters, in order to create cut-off values to determine the prognostic value of each parameter with regard to survival. Correlation analysis was performed calculating Pearson's correlation coefficient (*r*). A *P*-value <0.05 was considered statistically significant. Concerning symbolism and higher orders of significance: *P* < 0.05: *, *P* < 0.01: **, *P* < 0.001: ***, n.s.: not statistically significant, Ø: no data available.

## 3. Results

Demographic data and baseline clinical data of all study groups are presented in detail in Table 1. The primary site of infection in the septic group (double naming feasible) was the gastrointestinal tract, followed by the surgical site, and the respiratory tract (Table 1). Surgical site infections (SSI) were described to be source deep or organ space SSIs (e.g., organ space abscesses, insufficiencies of anastomoses, etc.) in all cases. Hospital-acquired pneumonia (HAP) or ventilator-associated pneumonia (VAP) due to several triggers (e.g., aspiration, insufficient coughing with subsequent retention of secretion, etc.) are thought to represent the most important reasons for a pulmonary site of infection. Since all patients of the septic cohort underwent a surgical procedure and were, therefore, hospitalized for a certain time prior to sepsis onset, community-acquired pneumonia (CAP) can most probably be disregarded as a pulmonary focus. However, results of microbiological diagnostics in tracheal secretion have not been recorded, so that these reflections cannot be validated. All patients with septic shock revealed cardiovascular failure with the need for catecholaminergic support. The incidence of further sepsis-associated organ failures is presented in Table 1. In contrast, no patients in the surgical group developed any organ failure, such as acute renal failure (ARF), acute respiratory distress syndrome (ARDS), or ALF. In the septic group, 38 of 60 patients (63.3%) survived 28 days. Patients who survived or died showed no significant differences concerning their demographic data (data not shown). None in the healthy or surgical groups died during the study.

**Figure 1:**
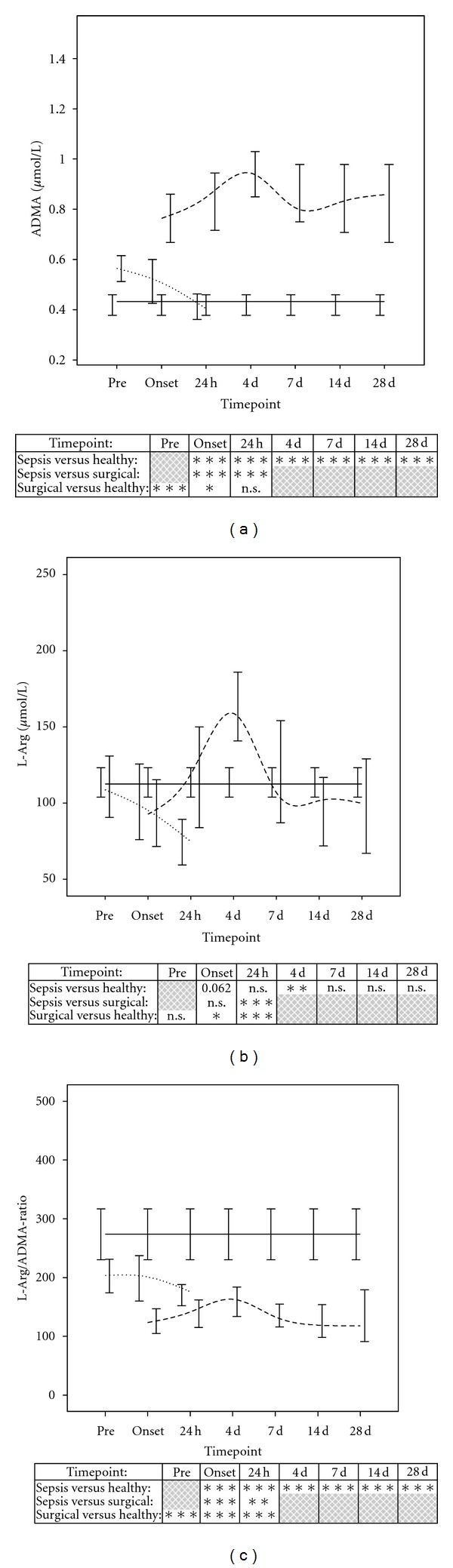
Comparison of ADMA (a) and L-arg (b) measurements as well as the resulting ratio of both (c) in healthy volunteers (*n* = 30, black continuous line), patients following major abdominal surgery (*n* = 30, short dashed line) and patients with septic shock (*n* = 60, long dashed line). Data in line charts are given as medians and the 95% CI. Concerning symbolism and higher orders of significance: *P* < 0.05: *, *P* < 0.01: **, *P* < 0.001: ***, n.s.: not statistically significant.

In healthy volunteers, the following plasma levels could be observed: ADMA: 0.433 *μ*mol/L; 0.369–0.506 *μ*mol/L, L-arg: 112.6 *μ*mol/L; 100.8–133.0 *μ*mol/L, Ratio L-arg/ADMA: 273.5; 218.8–329.3 (Median; Q1–Q3). In surgical patients, plasma levels of ADMA were significantly elevated prior to the surgical procedure. Postsurgery, ADMA plasma levels decreased towards the levels observed for the healthy volunteers (Figure 1(a)). Plasma levels of L-arg were comparable in healthy volunteers and surgical patients prior to the surgical procedure. Within 24 hours postsurgery, plasma levels of L-arg decreased and showed significant differences in comparison to healthy volunteers (Figure 1(b)). The ratio of L-arg and ADMA was significantly reduced in surgical patients at all timepoints (Figure 1(c)). When comparing subgroups of patients in the surgical group who did (*n* = 7) or did not (*n* = 23) receive a liver resection, no significant differences were observed in the plasma levels of L-arg, ADMA as well as the resulting ratio of both (data not shown).

In patients with septic shock, plasma levels of ADMA and L-arg showed comparable trends within the 28-day observation period with significantly increasing levels until 4 days after the onset of sepsis, followed by a sudden decrease until day 7 (Figures 1(a) and 1(b)). When septic patients were compared with healthy volunteers, plasma levels of ADMA were significantly elevated at all time points (Figure 1(a)). Analogous to the peak concentrations of ADMA, plasma levels of L-arg were also significantly elevated 4 days after sepsis onset in comparison to healthy volunteers. In contrast to ADMA, plasma levels of L-arg tended to be decreased at sepsis onset (*P* = 0.062) and showed no significant differences at the other timepoints within the 28-day observation period (Figure 1(b)). The resulting ratio of L-arg and ADMA in septic patients was significantly reduced in comparison to healthy volunteers as well as surgical patients at all time points (Figure 1(c)).

**Figure 2:**
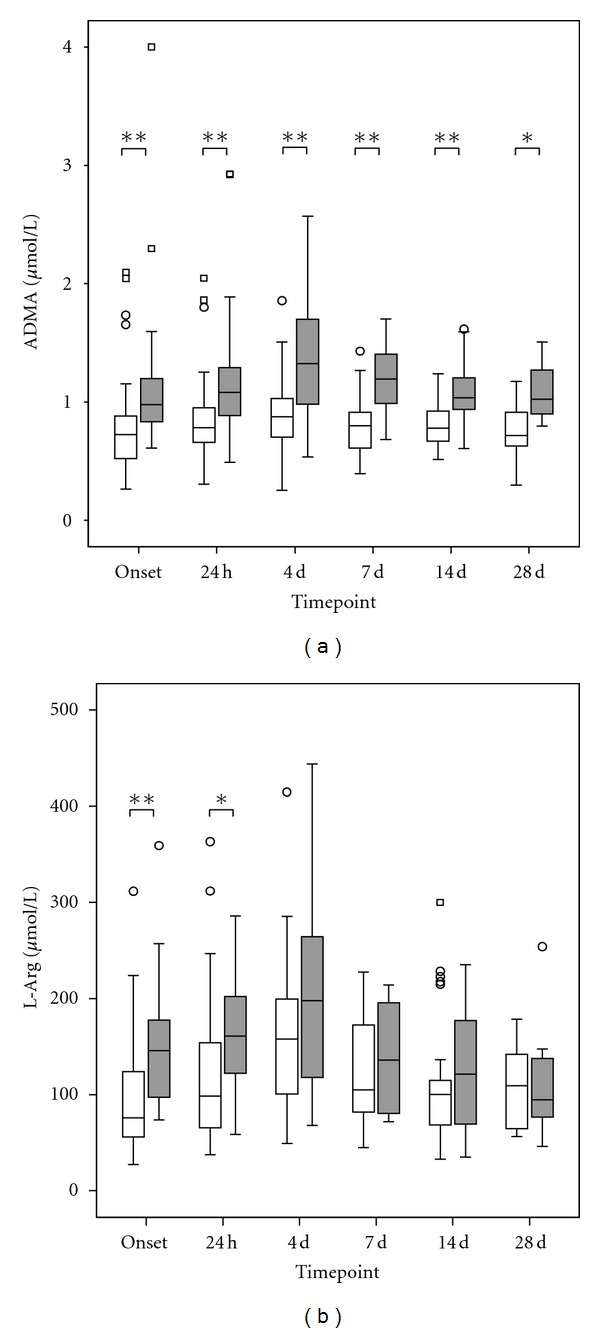
Comparison of ADMA (a) and L-arg (b) measurements in plasma samples of septic patients with an ALF (*n* = 15, grey box) in comparison to septic patients with a preserved liver function (*n* = 45, white box). Data in box plots are given as median, 25th percentile, 75th percentile, and the 1.5 interquartile range. Outliers are shown in form of circles (1.5–3 interquartile ranges above 75th percentile or below 25th percentile) or rectangles (>3 interquartile ranges above 75th percentile or below 25th percentile). Concerning symbolism and higher orders of significance: *P* < 0.05: *, *P* < 0.01: **, *P* < 0.001: ***, n.s.: not statistically significant.

When comparing subgroups of patients in the septic group who did or did not survive, plasma levels of ADMA tended to be increased at sepsis onset in the nonsurviving subgroup (*P* = 0.059). Afterwards, ADMA plasma levels were comparable between survivors and nonsurvivors. Plasma levels of L-arg were significantly elevated in the non-surviving subgroup 24 hours as well as 7 days after sepsis onset (Table 2). Results of subgroup analyses comparing septic patients with an ARDS versus non-ARDS or ARF versus non-ARF are further described in Table 2. Measurements of ADMA and L-arg plasma levels in patients with sepsis who did or did not develop sepsis-induced ALF (serum-bilirubin >/<4.0 mg/dL or 70.0 mmol/L [22]) are shown in Figures 2(a) and 2(b). ADMA was significantly elevated at all time points in the subgroup of ALF patients (Figure 2(a)). L-arg plasma levels were also significantly increased at sepsis onset and 24 hours later (Figure 2(b)), but not at later times. When comparing septic patients with an ALF who did (*n* = 8) or did not survive (*n* = 7) 28 days, plasma levels of ADMA showed a considerable trend towards higher levels in nonsurvivors at sepsis onset (*P* = 0.072). Afterwards ADMA plasma levels were comparable between the surviving and nonsurviving subgroup. In contrast, plasma levels of L-arg were significantly elevated in the nonsurviving subgroup at sepsis onset (*P* = 0.029*) but failed scarcely to show a significant difference 24 hours later (*P* = 0.054). At later time points, L-arg plasma levels were comparable between the surviving and nonsurviving subgroup. A comparable leveling could be observed for different routine markers for liver impairment. For example, plasma levels of lactate dehydrogenase (LDH) were significantly elevated in the nonsurviving subgroup at sepsis onset as well as 24 hours later. At later timepoints, LDH plasma levels were comparable between the surviving and nonsurviving subgroup. Substantially, similar results could be obtained for aspartat amino transferase (ASAT), alanine amino transferase (ALAT), as well as total bilirubine (Table 3).

**Figure 3:**
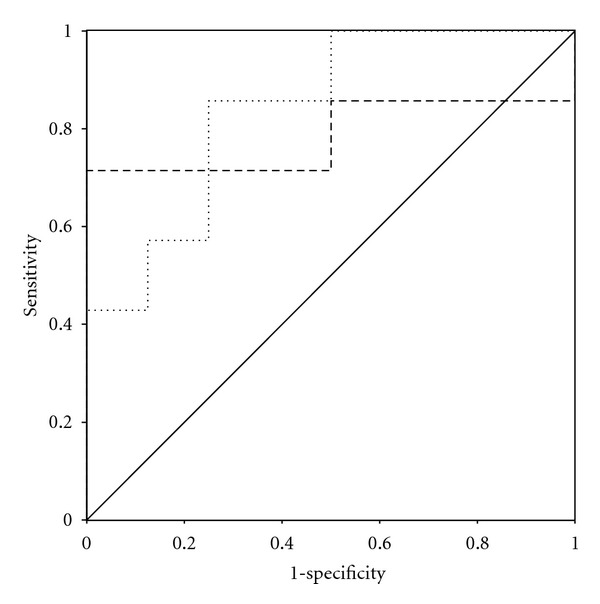
ROC curve for ADMA (long dashed line) and L-arg (short dashed line) plasma levels at sepsis onset in patients with septic shock and an accompanying ALF who ultimately did (*n* = 8) and did not (*n* = 7) survive within the 28 days observation period.

In septic patients with an ALF, ROC curve analysis revealed a cut-off value for L-arg at sepsis onset (area under the curve/AUC = 0.839) of 143.41 *μ*mol/L for early discrimination of survivors and nonsurvivors with a sensitivity of 0.857 and 1-specificity of 0.250 (Figure 3). Moreover, ROC analysis revealed a cut-off value for ADMA at the onset of sepsis syndrome (AUC = 0.786) of 0.853 *μ*mol/L with a sensitivity of 0.857 and 1-specificity of 0.500 (Figure 3).

In order to assess the influence of packed red blood cell (PRBC) administration on plasma levels of L-arg and ADMA, septic patients receiving PRBC <24 h prior to the respective blood draw (*n* = 39) were compared with septic patients not receiving PRBC <24 h prior to the respective blood draw (*n* = 21). At early (Onset, 24 h) as well as late stages of septic shock, L-arg and ADMA plasma levels did not differ significantly between the two subgroups (data not shown). Contrary to our expectations, in the interim phase (4 d) plasma levels of L-arginine and ADMA were significantly elevated in patients receiving PRBC (L-arg: *P* = 0.032*/ADMA: *P* = 0.028*). At this timepoint (4 d), septic patients receiving PRBC were shown to be more severely injured as assessed by disease severity scoring (acute physiology and chronic health evaluation (APACHE) II-score: 0.007**/SOFA-score: 0.037*/simplified acute physiology score (SAPS): 0.007**). Moreover, the influence of body's own red blood cells (RBCs) on plasma levels of L-arg and ADMA was assessed. Therefore, the amount of red cell mass in each patient at the different timepoints was estimated with the accompanying hemoglobin (Hb) concentration. Hb concentrations were shown to be significantly reduced in patients with septic shock as well as surgical patients in comparison to healthy volunteers at all timepoints (data not shown). An ensuing correlation analysis including all studygroups revealed that L-arg plasma levels were not correlated with the accompanying Hb concentrations (*r* = 0.090). By analogy, ADMA plasma levels showed only a weak correlation with Hb concentrations (*r* = 0.325).

## 4. Discussion

As assessed by plasma levels of ADMA and L-arg, the present investigation describes decreased endothelial NO bioavailability in patients with septic shock in comparison to healthy volunteers and patients following major abdominal surgery. Moreover, plasma levels of ADMA and L-arg were shown to be early predictors for survival in patients with septic shock and an accompanying ALF.

Concerning plasma levels of L-arg in septic patients, a recent review by Davis and Anstey failed to reveal conclusive results for patients with surgery-associated or trauma-associated sepsis [27]. In contrast, patients with sepsis not associated with trauma or surgery were observed to suffer from a hypoarginineaemic state [27]. Since our study was performed with patients suffering from septic shock on a surgical intensive care unit, the pathogenesis of sepsis was at least surgery-associated in all cases. L-arg plasma levels tended to be decreased in the initial phase of NO depletion, probably due to a lower *de novo* L-arg production (e.g., decreased food intake and gut absorption) as well as an increased L-arg consumption (e.g., increased arginase and NO production and elevated protein need) [12, 16]. This consuming phase was followed by a secondary short-term restorative phase with increasing plasma levels of L-arg at day 4. Comparable plasma kinetics have already been described by Davis et al., showing lowest L-arg plasma levels at sepsis onset followed by an increase 2–4 days later [28]. We hypothesize that this excess supply of L-arg should counteract increasing plasma levels of nonselective endogenous inhibitors such as ADMA, which are able to reduce the activity of NOS. The NO-reducing effects of ADMA have been proven in animal as well as human models using experimental ADMA-infusion [29, 30]. ADMA can also compete with L-arg, symmetrical dimethylarginine (SDMA) and L-lysine for the cationic amino acid transporters-dependant transport across the cell membrane, especially in hepatocytes [31]. While the ADMA uptake is performed for degradation, L-arg is used for NO-synthesis. Elevated plasma levels of ADMA have been reported for several disease states, which are known to be associated with an endothelial dysfunction such as hypertension, peripheral arterial disease, chronic renal disease, hypercholesterolemia, diabetes mellitus, and hyperhomocysteinemia [32–37]. Concerning patients with septic shock, ADMA plasma levels were also reported to be significantly elevated [2–4, 38, 39]. In contrast, in septic patients without shock, plasma ADMA concentrations did not differ compared to plasma levels in control patients [40]. As expected and in line with the literature, we were able to show significantly increased plasma levels of ADMA in the septic group of patients at all time points. Moreover, in analogy to the previously described results of L-arg measurements, a peak concentration for ADMA could be observed 4 days after sepsis onset. As described earlier, this comparable leveling of both, L-arg and ADMA may further support the hypothesis of the so-called L-arginine-paradox [21, 41]. Moreover, ADMA concentrations were shown to be elevated in patients of the surgical group prior to the surgical procedure in comparison to healthy volunteers. This effect might be due to several pre-existing conditions (e.g., hypertension, hypercholesterolemia) of the surgical cohort of patients, which are known to be associated with increased plasma levels of ADMA. Decreasing plasma levels of ADMA following the surgical procedure have most likely to be ascribed to simple dilution effects due to perioperative volume therapy.

With regard to the plasma levels of ADMA, the possible elimination pathways have to be taken into account, since MODS can frequently be observed in patients with septic shock [1]. ADMA is removed from the body by urinary excretion and therefore depends on kidney function [42]. On the other hand, ADMA is subject to enzymatic degradation into citrulline and dimethylamine by the enzyme dimethylarginine dimethylaminohydrolase (DDAH), which is highly expressed in the liver [43–45]. Accordingly, ADMA levels were shown to be significantly increased in patients with septic shock and an accompanying ARF. However, these results might have been underestimated since hemodialysis, peritonealdialysis and hemodiafiltration are known to be able to reduce ADMA plasma levels [46]. With respect to liver integrity, Nijfeldt et al. demonstrated in an animal model that the liver is a key player in the absorption and degradation of ADMA [42]. This observation was further supported by investigations in human patients suffering from either decompensated liver cirrhosis, ALF, or sepsis-associated hepatic dysfunction, showing a close correlation between ADMA plasma levels and the degree of hepatic dysfunction [2, 19, 47, 48]. Accordingly, we observed significantly elevated plasma levels of ADMA in patients with a sepsis-associated ALF. In terms of the “L-arginine-paradox,” L-arg plasma levels were also significantly elevated. Moreover, both biomarkers were shown to predict survival in this cohort of patients. However, the prognostic value of both biomarkers in septic patients with a sepsis-associated ALF seems to be most likely a reflection of poor hepatic function. Whether increased plasma levels of ADMA contribute causatively to the decease in these patients cannot finally be concluded.

With regard to further influencing factors of L-arg and ADMA plasma levels, especially red blood cells came into the focus of interest. On the one hand, administration of PRBC is reported to be associated with L-arg consumption, since PRBCs contain relevant amounts of arginase [49, 50]. This enzyme is able to convert arginine to ornithine, resulting in a reduced bioavailability of this conditionally essential amino acid. Probably due to the vanishing low amount of PRBC administration in the presented patients with septic shock, PRBC-associated arginine consumption could not be observed. Instead, plasma levels of L-arg in patients receiving PRBCs were significantly elevated at 4 d. This might be probably attributable to significantly increased ADMA plasma levels in more severely injured septic patients receiving PRBCs especially at 4 d. Beside PRBCs, the body's own RBC might likewise be an influencing factor, since RBCs do also contain relevant amounts of L-arg metabolizing enzymes (e.g., arginase) as well as express amino acid transporters for the L-arg uptake [51, 52]. However, since hemoglobin concentrations in patients with septic shock did not reveal any significant changes within the 28 day observation period and L-arg plasma levels showed a sigmoidal leveling, red cell mass is unlikely to have affected L-arg plasma levels in a relevant manner. This hypothesis is further strengthened throughout the subsidiary performed correlation analysis, which was not able to show a correlation between hemoglobin concentrations and the accompanying L-arg plasma levels.

Notably, in the literature, normal plasma levels of L-arg in healthy persons are reported to be 70–80 *μ*mol/L as assessed by HPLC (*high performance liquid chromatography*) [53–55]. This is in contrast to our investigation, since baseline L-arg plasma levels in healthy volunteers were described to be 112.6 *μ*mol/L in median. Moreover, L-arg plasma levels of patients with septic shock within our investigation tended to be steadily increased in comparison to the available literature. However, the ELISA-based measurements of ADMA did not differ in a relevant manner in comparison to the traditional form of ADMA measurements using HPLC. To define whether an increased sensitivity of the newly developed ELISA-based method for L-arg determination in human plasma samples might explain the discrepancy is beyond the scope of this manuscript and needs to be studied in the future.

## 5. Conclusions

NO-bioavailability was demonstrated to be reduced in patients with septic shock. In comparison to healthy volunteers and patients following major abdominal surgery, patients with septic shock showed significantly increased levels of ADMA, as well as decreased levels of the ratio of L-arg and ADMA at all time points within the 28-day observation period. Moreover, plasma levels of ADMA and L-arg were significantly increased in septic patients with an ALF in comparison to those with an intact hepatic function. Most important, measurements of ADMA and L-arg at sepsis onset appeared to be early predictors for survival in septic patients with ALF.

## Figures and Tables

**Table 1:** Baseline data of 60 patients in the septic group, 30 patients in the surgical group and 30 individuals in the healthy group.

Septic Group (*n* = 60)	
*Demographic Data*	
Age, y	69 ± 12; 70; 64–76
Male sex	46 (76.7%)
ASA-Status: I; II; III; IV; V	1 (1.7%); 11 (18.3%); 29 (48.3%); 15 (25.0%); 1 (1.7%)

*Primary site of infection/septic focus (Double naming feasible)*	
Lung	12 (20.0%)
Gastrointestinal tract	32 (53.3%)
Genitourinary tract	6 (10.0%)
Surgical site	16 (26.7%)
Others	2 (3.3%)

*Septic complications/organ failures [Sepsis Onset—28 d]*	
Acute renal failure (ARF)	35 (58.3%)
Acute respiratory distress syndrome (ARDS)	49 (81.2%)
Acute liver failure (ALF)	15 (25.0%)

Surgical Group (*n* = 30)	

*Demographic Data*	
Age, y	61 ± 12; 62; 57–70
Male sex	16 (53.3%)
ASA-Status: I; II; III; IV; V	0 (0.0%); 9 (30.0%); 20 (66.7%); 1 (3.3%); 0 (0.0%)

*Site of surgery (Double naming feasible)*	
Liver	7 (23.3%)
Pancreas	11 (36.7%)
Gastro-intestinal	27 (90.0%)

Healthy Group (*n* = 30)	

*Demographic Data*	
Age	27 ± 6; 26; 24–28
Male sex	19 (63.3%)
ASA-Status: I; II; III; IV; V	21 (70.0%); 9 (30.0%); 0 (0.0%); 0 (0.0%); 0 (0.0%)

Data are presented by number (%) or by mean ± standard deviation, median and interquartile range (Q1–Q3). Abbreviations: ASA-Status, physical status classification system according to the American Society of Anesthesiologists; ARF, acute renal failure; ARDS, acute respiratory distress syndrome; ALF, acute liver failure.

**Table 2:** L-arg and ADMA measurements in different subgroups of patients with septic shock.

Parameters	Units	L-arg and ADMA measurements in different subgroups of patients with septic shock											
Timepoints		Onset	24 h			4 d		7 d		14 d		28 d	
Survival:		Survivors at Day 28 (italic parts *n* = 38) versus Non-Survivors at Day 28 (bold parts, *n* = 22)											

L-arg	[*μ*mol/L]	*77.9;* * 62.1–115.3*	n.s.	*107.1;* * 65.9–150.1*	*	*154.2; * *113.4–228.8*	n.s.	*97.0; * *76.5–154.8*	*	*98.4;* * 67.7–125.0*	n.s.	*99.8;* * 66.4–138.3*	Ø
		**123.7;** ** 66.1–175.4**		**158.4;** ** 88.2–201.5**		**197.5; ** **149.7–240.0**		**172.2; ** **161.8–195.9**		**112.1;** ** 102.1–145.3**		**Ø**	
ADMA	[*μ*mol/L]	*0.74;* * 0.52–0.92*	n.s.	*0.84*; * 0.67–1.08 *	n.s.	*0.95;* * 0.72–1.11*	n.s.	*0.80;* * 0.63–1.05*	n.s.	*0.83;* * 0.70–1.04*	n.s.	*0.86;* * 0.67–0.99*	Ø
		**0.83;** ** 0.61–1.21**		**0.87;** ** 0.67–1.29**		**0.87;** ** 0.71–1.27**		**0.83;** ** 0.77–1.14**		**0.75;** ** 0.60–0.91**		**Ø**	
L-arg/ADMA-Ratio	[none]	*126.5;* * 91.5–164.8*	n.s.	*135.0;* * 95.0–197.8*	n.s.	*154.0;* * 123.0–191.5*	n.s.	*121.5;* * 97.5–160.5*	*	*111.0;* * 83.5–155.8*	n.s.	*118.0;* * 89.5–180.5*	Ø
		**118.5;** ** 91.5–157.0**		**142.5;** ** 105.0–186.5**		**196.0;** ** 138.0–235.5**		**185.0;** ** 143.5–240.0**		**191.0;** ** 165.3–204.8**		**Ø**	

Pulmonary:	Patients with ARDS (italic parts, *n* = 49) versus Non-ARDS (bold parts, *n* = 11)												

L-arg	[*μ*mol/L]	*84.6;* * 61.4–145.9*	n.s.	*114.3;* * 72.9–161.0*	n.s.	*167.5;* * 132.6–242.1*	*	*106.8;* * 84.7–172.2*	n.s.	*102.9;* * 69.2–128.8*	n.s.	*120.0;* * 66.7–149.3*	n.s.
		**94.6;** ** 83.5–140.9**		**187.8;** ** 152.2–228.0**		**76.0;** ** 57.4–108.5**		**109.6;** ** 91.2–168.5**		**150.8;** ** 117.4–184.1**		**99.8;** ** 99.8-99.8**	
ADMA	[*μ*mol/L]	*0.81;* * 0.61–0.93*	n.s.	*0.85;* * 0.69–1.08*	n.s.	*1.00;* * 0.72–1.19*	n.s.	*0.80;* * 0.68–1.05*	n.s.	*0.82;* * 0.68–1.03*	n.s.	*0.85;* * 0.67–1.00*	n.s.
		**0.55;** ** 0.53–1.67**		**0.74;** ** 0.67–1.62**		**0.78;** **0.68–0.89**		**0.83;** ** 0.71–1.03**		**0.84; ** **0.78–0.91**		**0.90;** ** 0.90-0.90**	

L-arg/ADMA-Ratio	[none]	*124.0;* * 90.0–162.0*	n.s.	*141.0;* * 97.5–183.5*	n.s.	*172.5;* * 129.0–215.5*	n.s.	*137.0;* * 102.0–171.0*	n.s.	*120.0;* * 93.0–162.0*	n.s.	*128.0;* * 90.3–179.8*	n.s.
		**109.0;** ** 88.5–158.0**		**169.5; ** **107.5–324.5**		**111.0; ** **80.3–142.3**		**132.0;** ** 127.0–158.5**		**170.0;** ** 144.0–196.0**		**110.0; ** **110.0-110.0**	

Renal:	Patients with ARF (italic parts, *n* = 35) versus Non-ARF (grey boxes, *n* = 25)												

L-arg	[*μ*mol/L]	*95.8;* * 60.3–150.5*	n.s.	*114.3;* * 76.3–192.7*	n.s.	*185.9;* * 140.7–246.7*	*	*147.8;* * 86.7–180.9*	n.s.	*101.6;* * 69.0–146.6*	n.s.	*120.8;* * 65.6–147.6*	n.s.
		**87.8;** ** 67.0–142.7**		**134.7;** ** 72.6–160.4**		**142.1; ** **105.3–162.9**		**99.1; ** **75.9–117.0**		**96.5;** ** 68.1–111.6**		**81.6;** ** 69.7–96.3**	
ADMA	[*μ*mol/L]	*0.82;* * 0.59–1.09*	n.s.	*0.86;* *0.66–1.10*	n.s.	*1.02;* * 0.75–1.35*	*	*0.92;* * 0.75–1.26*	*	*0.85;* * 0.67–1.01*	n.s.	*0.85;* * 0.67–0.98*	n.s.
		**0.73;** ** 0.51–0.86**		**0.83; ** **0.67–1.03**		**0.87;** **0.68–1.01**		**0.79;** ** 0.59–0.85**		**0.83;** ** 0.70–1.06**		**0.91; ** **0.53–1.01**	
L-arg/ADMA-Ratio	[none]	*118.0;* * 95.0–160.5*	n.s.	*122.0;* * 98.0–179.0*	n.s.	*172.0;* * 129.0–196.0*	n.s.	*141.0;* * 105.5–169.5*	n.s.	*123.5; * *93.5–168.0*	n.s.	*138.0;* * 94.0–179.0*	n.s.
		**126.0;** ** 88.3–169.8**		**146.5;** ** 100.0–207.0**		**154.0; ** **109.0–230.0**		**120.5;** ** 97.5–188.8**		**100.0; ** **75.5–125.5**		**96.0; ** **65.5–172.3**	

Data are presented by median and interquartile range (Q1–Q3). Concerning symbolism and higher orders of significance: *P* < 0.05: *, *P* < 0.01: **, *P* < 0.001: ***, n.s.: not statistically significant, Ø: no data available. Abbreviations: L-arg, L-arginine; ADMA, asymmetric dimethylarginine; ARDS, acute respiratory distress syndrome; ARF, acute renal failure.

**Table 3:** Routine markers for liver impairment in surviving and nonsurviving septic patients with an ALF.

Parameters	Units	Routine markers for liver impairment in surviving and nonsurviving septic patients with an ALF.											
Timepoints		Onset		24 h		4 d		7 d		14 d		28 d	
ALF:		Survivors at Day 28 (italic parts, *n* = 8) versus nonsurvivors at Day 28 (bold parts, *n* = 7)											

ASAT	[U/L]	*48.0; * *23.0–132.0*	.072	*51.0; * *25.5–164.0*	.072	*62.0;* * 55.8–89.3*	n.s.	*82.5; * *47.8–115.3*	n.s.	*52.0; * *38.5–69.8*	n.s.	*44.0; * *39.5–99.5*	Ø
		**534.0; ** **56.0–1798.5**		**438.0;** ** 100.0–1653.0**		**113.0;** ** 71.0–621.0**		**505.5; ** **267.8–743.3**		**52.0; ** **43.0–61.0**		**Ø**	
ALAT	[U/L]	*21.0; * *13.8–43.5*	*	*20.0; * *14.0–47.8*	.054	*30.5;* * 19.8–39.5*	n.s.	*23.5; * *18.5–38.0*	n.s.	*26.5; * *18.5–45.0*	n.s.	*31.0; * *19.0–72.0*	Ø
		**266.0; ** **45.5–365.0**		**298.0; ** **50.0–846.0**		**125.0; ** **74.5–290.5**		**211.0; ** **116.5–305.5**		**49.5; ** ** 36.8–62.3**		**Ø**	
LDH	[U/L]	*203.0; * *190.8–255.5*	*	*188.5; * *179.5–230.5*	**	*229.0;* * 173.8–264.3*	n.s.	*237.5; * *187.5–327.8*	n.s.	*216.0;* * 196.3–232.5*	n.s.	*216.0; * *184.0–262.5*	Ø
		**453.0;** ** 271.0–1302.5**		**339.0; ** **300.0–1267.0**		**315.0; ** **279.0–968.0**		**664.0; ** **416.5–911.5**		**236.0;** ** 233.0–239.0**		**Ø**	
Total bilirubine	[mg/dL]	*4.0; * *1.9–4.5*	.054	*3.1; * *2.1–4.7*	.094	*3.8;* * 2.9–7.8*	n.s.	*4.7;* * 2.9–10.0*	n.s.	*5.8; * *3.4–13.1*	n.s.	*6.4; * *4.2–14.7*	Ø
		**5.8; ** **4.8–7.5**		**9.0; ** **4.2–9.6**		**2.4; ** **2.3–9.2**		**2.3; ** **2.2–2.5**		**4.8; ** **3.3–6.2**		**Ø**	

Data are presented by median and interquartile range (Q1–Q3). Concerning symbolism and higher orders of significance: *P* < 0.05: *, *P* < 0.01: **, *P* < 0.001: ***, n.s.: not statistically significant, Ø: no data available. Abbreviations: ALF, acute liver failure; ASAT, aspartat amino transferase; ALAT, alanine amino transferase; LDH, lactate dehydrogenase.

## Abbreviations

ADMA: Asymmetric dimethylarginine
ALAT: Alanine amino transferase
ALF: Acute liver failure
APACHE II-score: Acute physiology and chronic health evaluation II-score
ARF: Acute renal failure
ARDS: Acute respiratory distress syndrome
ASAT: Aspartat amino transferase
AUC: Area under the curve
CAP: Community acquired pneumonia
DDAH: Dimethylarginine dimethylaminohydrolase
ELISA: Enzyme linked immunosorbent assay
HAP: Hospital acquired pneumonia
Hb: Hemoglobin
HPLC: High performance liquid chromatography
L-arg: L-arginine
LDH: Lactate dehydrogenase
MODS: Multiple organ dysfunction syndrome
NO: Nitric oxide
NOS: Nitric oxide synthases
PRBC: Packed red blood cells
RBC: Red blood cells
ROC: Receiver operating characteristic
SAPS: Simplified acute physiology score
SDMA: Symmetrical dimethylarginine
SOFA-score: Sequential organ failure assessment-score
SPSS: Statistical product and services solutions
SSI: Surgical site infection
VAP: Ventilator associated pneumonia.
